# AQP3-Dependent PI3K/Akt Modulation in Breast Cancer Cells

**DOI:** 10.3390/ijms24098133

**Published:** 2023-05-01

**Authors:** Monika Mlinarić, Ivan Lučić, Lidija Milković, Inês V. da Silva, Ivana Tartaro Bujak, Vesna Musani, Graça Soveral, Ana Čipak Gašparović

**Affiliations:** 1 Division of Molecular Medicine, Ruđer Bošković Institute, 10000 Zagreb, Croatia; 2 Research Institute for Medicines (iMed.ULisboa), Faculty of Pharmacy, Universidade de Lisboa, 1649-003 Lisbon, Portugal

**Keywords:** AQP3, PI3K/Akt, NRF2

## Abstract

Aquaporin 3 (AQP3) is a peroxiporin, a membrane protein that channels hydrogen peroxide in addition to water and glycerol. AQP3 expression also correlates with tumor progression and malignancy and is, therefore, a potential target in breast cancer therapy. In addition, epithelial growth factor receptor (EGFR) plays an important role in breast cancer. Therefore, we investigated whether disruption of the lipid raft harboring EGFR could affect AQP3 expression, and conversely, whether AQP3 silencing would affect the EGFR/phosphoinositide-3-kinase (PI3K)/Protein kinase B (PKB or Akt) signaling pathway in breast cancer cell lines with different malignant capacities. We evaluated H_2_O_2_ uptake, cell migratory capacity, and expression of PI3K, pAkt/Akt in three breast cancer cell lines, MCF7, SkBr3, and SUM159PT, and in the nontumorigenic breast epithelial cell line MCF10A. Our results show different responses between the tested cell lines, especially when compared to the nontumorigenic cell line. Neither lipid raft disruption nor EGF stimuli had an effect on PI3K/Akt pathway in MCF10A cell line. AQP3-silencing in SkBr3 and SUM159PT showed that AQP3 can modulate PI3K/Akt activation in these cells. Interestingly, SUM159PT cells increase nuclear factor-E2–related factor 2 (NRF2) in response to lipid raft disruption and EGF stimuli, suggesting an oxidative-dependent response to these treatments. These results suggest that in breast cancer cell lines, AQP3 is not directly related to PI3K/Akt pathway but rather in a cell-line-dependent manner.

## 1. Introduction

Breast cancer is still associated with high mortality due to metastases [1]. There are numerous factors that contribute to the breast cancer mortality and metastasis. In fact, new molecules that affect signaling pathways and contribute to tumor malignancy are being constantly described. Aquaporins (AQPs), initially discovered as water channels, are pointed out as players in cancer biology with a crucial role in cellular processes and signaling pathways activation [2,3].

These membrane pores regulate the fluxes of water, glycerol, hydrogen peroxide, and other small molecules [4,5]. They are a family of membrane channels consisting of 13 family members (AQP0-AQP12) in mammals, with selectivity for different substrates and different cellular locations [6,7]. Aquaporins are generally divided into three subgroups according to their pore selectivity and primary structure: orthodox AQPs (AQP0, AQP1, AQP2, AQP4, AQP5, AQP6, AQP8) which primarily transport water; aquaglyceroporins (AQP3, AQP7, AQP9, and AQP10) which also transport glycerol and other small neutral solutes; and S-aquaporins (AQP11 and AQP12) which are localized only intracellularly [4]. In addition to water and glycerol, aquaporins can permeate a variety of different small polar molecules, among which hydrogen peroxide (H_2_O_2_) is receiving increasing attention [8]. The importance of channeling H_2_O_2_ is reflected in their grouping into a fourth group named peroxiporins based on their ability to permeate H_2_O_2_, and includes aquaporins from the other three groups (AQP1, AQP3, AQP5, AQP8, AQP9, and AQP11). Interestingly, peroxiporins are elevated in tumors, and are associated with malignant forms [9,10,11]. In particular, AQP3 has been mostly associated with cancer progression and metastasis and is suggested to have high potential as a therapeutic target for breast cancer [12,13].

**Figure 1:**
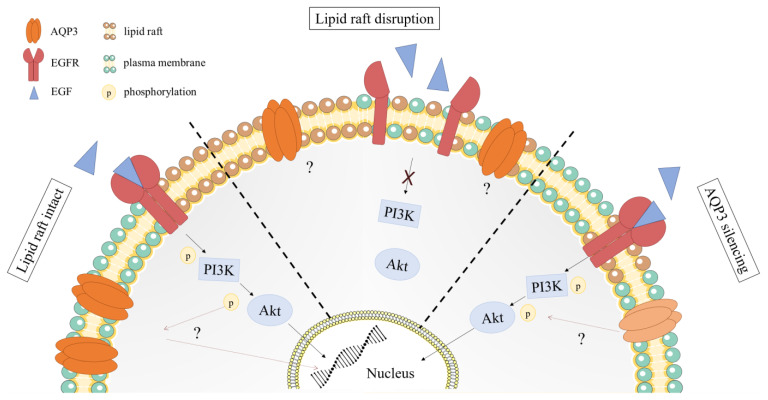
Working hypothesis. (1) Lipid raft intact: EGFR are located in lipid rafts and dimerize upon binding of EGF; dimerized EGFR starts signaling cascade of phosphorylation of PI3K and Akt, making the pathway active. How does the activation of the EGFR/PI3K/akt pathway influence the AQP3 activity? (2) Lipid raft disruption: cholesterol depletion disrupts lipid rafts so EGFR cannot dimerize and activate PI3K/Akt pathway. Are AQP3 expression and activity affected by lipid raft disruption? (3) AQP3 silencing: How does AQP3 silencing affect EGFR/PI3K/Akt pathway?

The regulation of aquaporins and their involvement in signaling pathways is still not entirely understood [14]. Studies suggest that the PI3K/Akt (phosphoinositide-3-kinase/protein kinase B) signaling pathway is involved in the regulation of AQP3 expression in human keratinocytes when treated with Poria cocos extract [15]. Other studies suggest that AQP3 expression is upregulated by EGF (epidermal growth factor) through the MAPK/ERK (mitogen-activated protein kinase/extracellular signal-regulated kinase) pathway [16]. In human gastric carcinoma cells, AQP3 increases matrix metalloproteinases via a PI3K/Akt-dependent manner [17]. AQP3 also regulates EGFR signaling by modulating H_2_O_2_ transport to the cells, and knockdown of AQP3 impairs EGFR signaling pathway [12]. Moreover, AQP3 is located near the H_2_O_2_-producing NADPH oxidase 2 (NOX2) and transports H_2_O_2_ produced by NOX2 into the cancer cells [18]. We still do not understand the link between H_2_O_2_ transport through aquaporins and the tumor malignancy, nor with the therapy resistance, but a study on colon cancer cells correlated the higher expression of AQP3 and AQP5 with the resistance to H_2_O_2_-induced stress [19]. Since studies suggest an interplay between EGFR signaling and AQP3, we aimed to investigate whether AQP3 is localized near the EGF receptor, which is known to localize within lipid rafts [20,21]. We also hypothesized that AQP3 expression could be affected by lipid rafts disruption via causing impaired EGFR signaling (Figure 1). To ensure that this interaction is not the other way around, we silenced AQP3 of breast cancer cells and measured activation of the EGFR/PI3K/Akt pathway. Our results suggest that AQP3 modulates PI3K/Akt activation and oxidative response in cancer cells, and that this modulation is cell-line-specific. Furthermore, AQP3 may indirectly contribute to the oxidative resistance of the SUM159PT cancer cell line, prompting future studies to investigate AQP3 regulation of cancer stress resistance.

## 2. Results

To investigate if EGFR is related to AQP3 in breast cancer, we used three different types of breast cancer cell lines, the estrogen receptor (ER)- and progesterone receptor (PR)-positive cell line, MCF7, the HER2-positive cell line, SkBr3, and the ER-, PR-, and HER2-negative (triple negative) cell line, SUM159PT. As a control, the nontumorigenic breast epithelial cell line, MCF10A, was used.

### 2.1. Effects of Lipid Raft Disruption and EGF on Cell Viability and Proliferation

**Figure 2:**
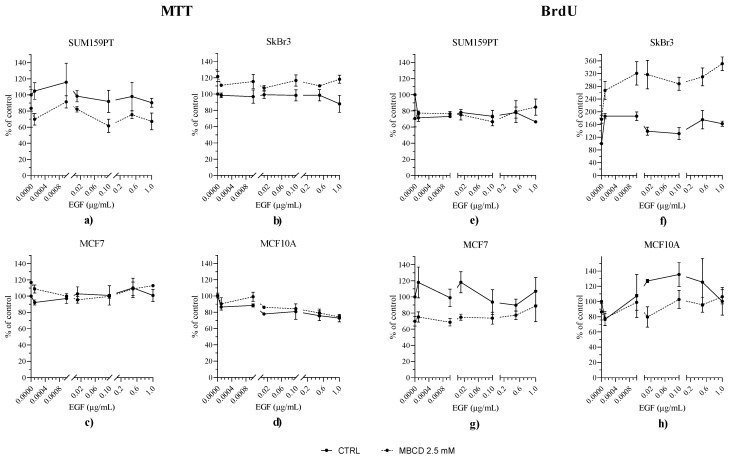
Effect of EGF stimuli on viability and proliferation of MBCD-treated cells. SUM159PT, SkBr3, MCF7, and MCF10A were treated with 2.5 mM MBCD for 2 h, when the media were changed and cells were treated with EGF in a range of concentrations. After 24 h, MTT or BrdU assays were performed. Panels (**a**) SUM159PT, (**b**) SkBr3, (**c**) MCF7, (**d**) MCF10A cells tested for viability (MTT assay); panels (**e**) SUM159PT, (**f**) SkBr3, (**g**) MCF7, (**h**) MCF10A tested for cell proliferation (BrdU incorporation assay). Experiments were performed in biological and technical triplicates. Cell viability and proliferation were calculated as the ratio between treated cell and control for each treatment, multiplied by 100 (% of control), and are presented as mean ± SEM.

Firstly, we determined the incubation time and the effective concentration of methyl-beta-cyclodextrin (MBCD) and EGF by testing the range of concentrations of these two compounds (results shown in Supplemental Figures S1–S3). Next, we tested the effects of lipid raft disruption with 2.5 mM MBCD in combination with EGF stimulation on cell viability and proliferation. Cell viability was evaluated by MTT assay (Figure 2a–d) and proliferation by BrdU incorporation assay (Figure 2e–h) in order to determine the effective concentration of each compound. EGF treatment slightly, but not significantly, decreased the viability of SUM159PT cells, and had no effects on their proliferation. EGF stimuli increased the viability and proliferation of SkBr3 cells (*p* < 0.0001). EGF had no effect on the viability of MCF7 cells, while it decreased proliferation in combination with MBCD. EGF treatment did not have any effect on MCF10A cells viability or proliferation.

### 2.2. Effects of Lipid Raft Disruption and EGF on the Peroxiporin Activity

**Figure 3:**
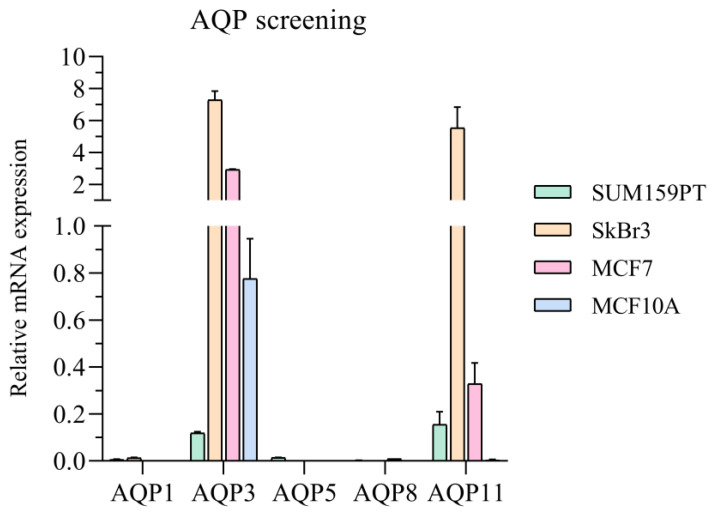
Screening for the basal mRNA expression of AQP1, AQP3, AQP5, AQP8, and AQP11 in SUM159PT, SkBr3, MCF7, and MCF10A cell lines. Cells were grown till 80% confluency, when the RNA was isolated and analyzed by qPCR. Results are presented as mean ± SEM.

In order to confirm the presence of AQP3 in the breast cancer cell lines, we performed screening for several aquaporin isoforms. These results showed that of the selected isoforms, AQP3 and AQP11 were present in all three breast cancer cell lines, and only AQP3 was present in the MCF10A cell line, the nontumorigenic cell line (Figure 3). Therefore, we further investigated the correlation of AQP3 with the PI3K/Akt pathway which was reported as dysregulated in cancer.

**Figure 4:**
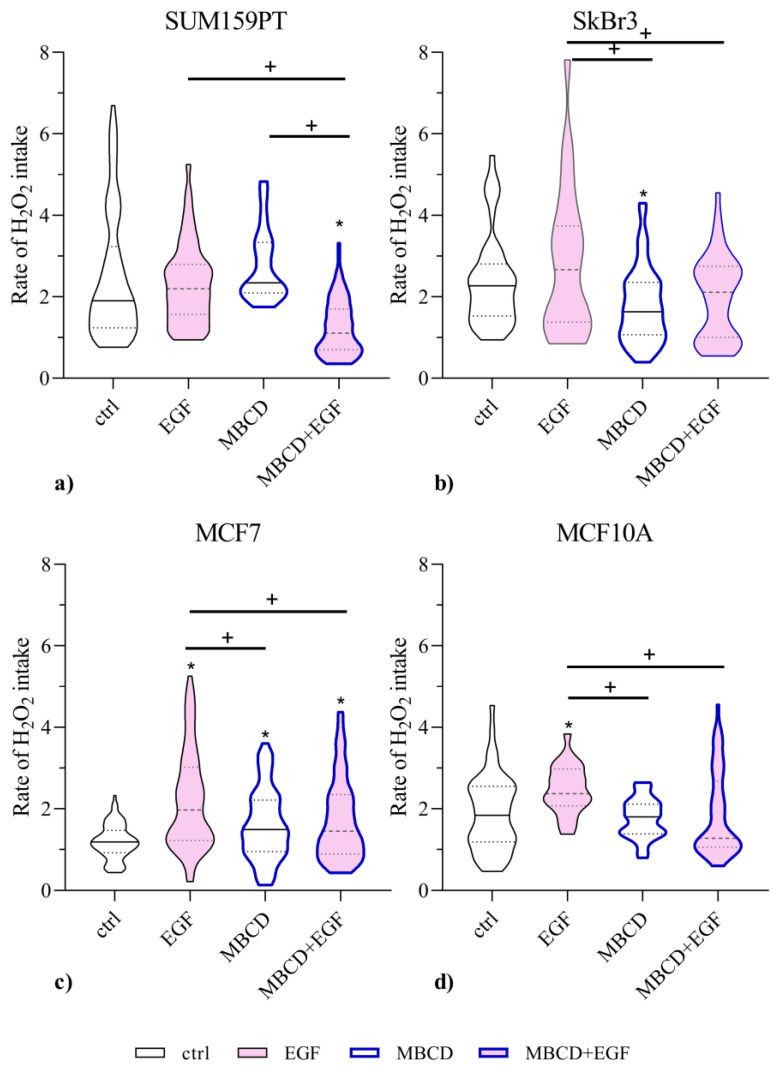
Effect of EGF stimuli and MBCD treatment on peroxiporin activity. Panels (**a**) SUM159PT, (**b**) SkBr3, (**c**) MCF7, and (**d**) MCF10A were treated with 2.5 mM MBCD for 2 h, when the media were changed and cells were treated with 0.1 µg/mL EGF. After 24 h, cells were assayed for H_2_O_2_ intake. Data present first-order kinetic rate constant (s−1) of H_2_O_2_ influx through peroxiporins after addition of 100 µM H_2_O_2_. The results of biological triplicates are shown as violin plots showing mean (central line) with interquartile range (upper and lower lines) together with the distribution of the data (shape of plots). * *p* < 0.05 compared to control (untreated); + *p* < 0.05 compared to other groups.

Previously, we showed that AQP3 has the ability to channel H_2_O_2_ to into the pancreatic cell line BxPC-3 [22,23]. Therefore, to assess if lipid raft disruption and EGF treatment affect peroxiporin activity, we evaluated H_2_O_2_ membrane permeability by measuring the rate of ROS accumulation due to H_2_O_2_ influx in breast cancer cells. Figure 4 displays the data obtained in single cell analysis for each condition, where we analyzed at least 25 cells in each biological triplicate (total N = 75). In SUM159PT cells, H_2_O_2_ intake rate was significantly lower in SUM159PT cells treated with both MBCD and EGF compared to control group, and groups treated with EGF or MBCD alone (all *p* < 0.0001). SkBr3 cells treated with MBCD alone also showed decreased peroxiporin activity compared to control or EGF (*p* = 0.047 or *p* = 0.002, respectively). Although EGF treatment after exposure to MBCD increased peroxiporin activity, it could not achieve the activity of EGF treatment alone (*p* = 0.0217). MCF7 cells showed differential response of aquaporin activity, and all treatments resulted in increased activity compared to control (EGF *p* < 0.0001, MBCD *p* = 0.0091, and MBCD + EGF *p* = 0.0004). Interestingly, MBCD and MBCD in combination with EGF decreased aquaporin activity compared to EGF treatment alone (*p* < 0.0001 and *p* = 0.0018, respectively). In MCF10A cells, EGF increased aquaporin activity compared to all groups (vs. control *p* = 0.036, vs. MBCD *p* = 0.0243, vs. MBCD + EGF *p* = 0.0253).

### 2.3. Effects of Lipid Raft Disruption and EGF on Cell Migration

**Figure 5:**
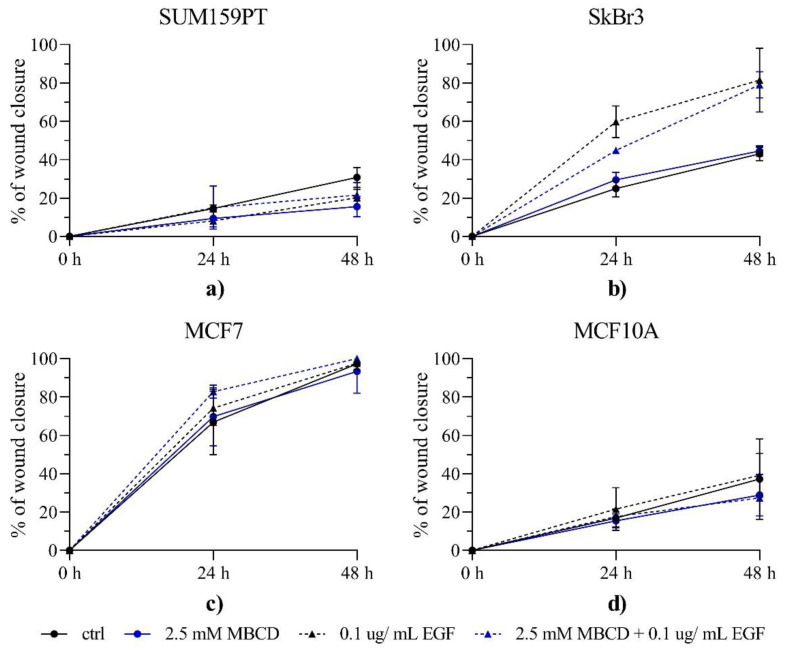
Effect of EGF stimuli and MBCD treatment on cell migration. Panels (**a**) SUM159PT, (**b**) SkBr3, (**c**) MCF7, and (**d**) MCF10A cells were treated with 2.5 mM MBCD for 2 h, when the media were changed and cells were treated with 0.1 µg/mL EGF. Cells were photographed after scratching (0 h), after 24 h, and 48 h, and analyzed by ImageJ software 1.53t. Experiments were performed in biological and technical triplicates. Results are presented as mean ± SEM.

Aquaporins have been reported to affect cancer cell migration by possibly inducing the redistribution of water fluxes in the cellular membrane [23,24], altering cell–cell adhesion and modulating cell biomechanical properties [22,25]. Therefore, we evaluated the impact of MBCD and EGF treatments on cell migration by the wound healing assay, performed with mitomycin C, which inhibits proliferation (Figure 5). SUM159PT cells showed the slowest rate of wound closure, with no statistical differences between groups, except for a decrease in migration of the MBCD-treated group compared to control after 48 h (*p* < 0.0014). Wound closure in SkBr3 cells was significantly increased by EGF treatment compared to control, and it remained significantly increased at both time points (*p* < 0.0001 for both). The MBCD treatment did not change the percentage of wound closure compared to control. Cells treated with MBCD prior to EGF treatment showed decreased wound closure (*p* = 0.0412) after 24 h compared to EGF treated cells, but not after 48 h. Interestingly, neither MCF7 nor MCF10A cells showed any differences between treatments, and MCF7 cells showed the highest ability to close the wound of all cells tested.

### 2.4. Effects of Lipid Raft Disruption and EGF on Protein Expression

**Figure 6:**
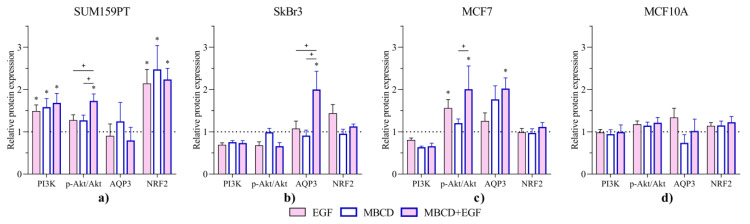
Effect of EGF stimuli and MBCD treatment on the expression of PI3K, pAkt/Akt, AQP3, and NRF2. (**a**) SUM159PT, (**b**) SkBr3, (**c**) MCF7, and (**d**) MCF10A. Cells were treated with 2.5 mM MBCD for 2 h, when the media were changed and cells were treated with 0.1 µg/mL EGF. After 24 h, cells were harvested and Western blot was performed to study the expression of the selected proteins. The protein levels are presented as relative values compared to control, which is presented as dashed line. Experiments were performed in biological and technical triplicates. Results are presented as mean ± SEM. * *p* < 0.05 compared to control (untreated); + *p* < 0.05 compared to other groups.

We analyzed the expression of AQP3, PI3K, and pAkt/Akt as a part of EGFR/PI3K/Akt pathway, as well as NRF2 in three cancer cell lines and nontumorigenic cell line in order to investigate the influence of lipid raft disruption and EGF treatment on these proteins (Figure 6). Interestingly, neither MBCD nor EGF affected any of the target proteins in the nontumorigenic MCF10A cell line. Contrary, significant changes were observed for each cancer cell line. Expression of PI3K was significantly increased in SUM159PT cells in all treatments (ctrl vs. EGF *p* = 0.0033, MBCD vs. ctrl *p* = 0.0005, MBCD + EGF vs. ctrl *p* < 0.0001) compared to control. Interestingly, the activation of Akt, via the ratio pAkt/Akt, did not follow the pattern of PI3K in SUM159PT. While the pAkt/Akt was unchanged in SkBr3 and MCF10A cells regardless of the treatment, in SUM159PT it was significantly increased after MBCD and EGF treatment compared to control and to MBCD and EGF treatment alone (*p* = 0.0001, *p* = 0.0331, and *p* = 0.0302, respectively). In MCF7 cells, Akt is significantly activated upon EGF treatment regardless of MBCD (compared to control *p* = 0.0499 and *p* < 0.0001, respectively, and MBCD vs. MBCD + EGF *p* = 0.0042). AQP3 expression did not change in SUM159PT and MCF10A cells, while it increased in SkBr3 cells treated with MBCD + EGF compared to all groups (vs. ctrl *p* = 0.0113, vs. EGF *p*= 0.0223, and vs. MBCD *p* = 0.0074). MCF7 cells showed similar pattern for AQP3, but with significant difference only to untreated control (*p* = 0.043). Finally, NRF2 expression changed only in SUM159PT cells, showing significant increase in all treatment groups compared to control (*p* < 0.0001 for all groups).

### 2.5. Effects of Silenced AQP3 on PI3K/Akt Pathway

**Figure 7:**
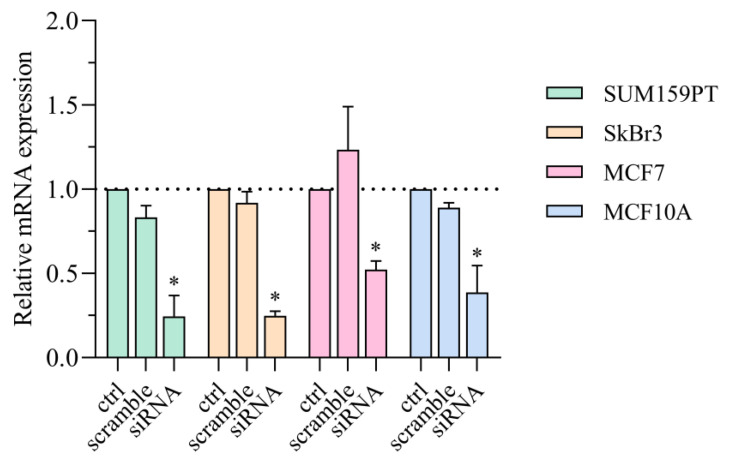
Effect of AQP3 silencing in SUM159PT, SkBr3, MCF7, and MCF10A cell lines assayed by Western blot. Cells were treated with siAQP3 or control (scrambled) siRNA 72 h for protein expression. Experiments were performed in biological and technical triplicates. Results are presented as mean ± SEM. * *p* < 0.05 compared to control (ctrl).

In order to study the effect of lipid raft disruption and EGF stimuli together with the relation between AQP3 and PI3K/Akt signaling pathway, we silenced AQP3 gene expression. The effectiveness of AQP3 silencing on mRNA and protein levels is represented in Figure 7, showing that AQP3 gene and protein expressions were significantly decreased in all cell lines.

**Figure 8:**
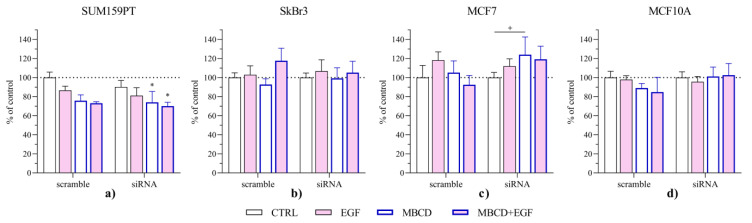
Effect of AQP3 silencing and EGF stimuli and MBCD treatment on the viability of (**a**) SUM159PT, (**b**) SkBr3, (**c**) MCF7, and (**d**) MCF10A. Cells were treated with siAQP3 or control (scrambled) siRNA for 72 h after which they were treated with 2.5 mM MBCD for 2 h, when the media were changed and cells were treated with 0.1 µg/mL EGF. After 24 h, cells were assayed for viability by MTT assay. The viability of each group is presented as relative values compared to control cells (untreated and without siRNA), which is shown as dashed line. Experiments were performed in biological and technical triplicates. Results are presented as mean ± SEM. * *p* < 0.05 compared to control (untreated cells with scrambled siRNA), + *p* < 0.05 compared to other groups.

**Figure 9:**
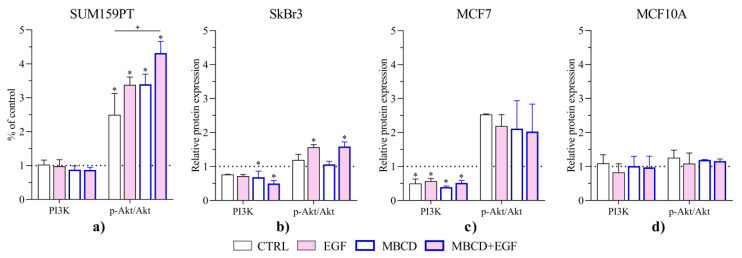
Effect of AQP3 silencing, EGF stimuli, and MBCD treatment on the expression of PI3K and pAkt/Akt of (**a**) SUM159PT, (**b**) SkBr3, (**c**) MCF7, and (**d**) MCF10A. Cells were treated with siAQP3 or control (scrambled) siRNA for 72 h when the media were exchanged with 2.5 mM MBCD for 2 h, after which cells were treated with 0.1 µg/mL EGF for 24 h. Cells were harvested and Western blot was performed to study the expression of the selected proteins. Experiments were performed in biological and technical triplicates. Protein levels are presented as relative values compared to the control (untreated and with scrambled siRNA), which is shown as dashed line. Results are presented as mean ± SEM. * *p* < 0.05 compared to control (untreated cells with scrambled siRNA), + *p* < 0.05 compared to other groups.

We then investigated the effect of AQP3 silencing and treatments on cell viability (Figure 8) and the expression of AQP3, PI3K, and pAkt/Akt as a part of EGFR/PI3K/Akt pathway (Figure 9). Interestingly, in SkBr3 cells, which had the highest expression of AQP3, silencing did not change viability when treated with EGF, MBCD, or in combination. The same was observed in MCF10A cells, while SUM159PT showed the opposite pattern to MCF7 cells. In SUM159PT cells, viability was significantly decreased by MBCD both alone and with EGF. In MCF7 cells, MBCD alone significantly increased viability, while in combination with EGF this increase was not significant.

Finally, we evaluated the effect of AQP3 silencing on PI3K and pAkt/Akt protein expression (Figure 9). AQP3-silenced MCF10A showed similar results to the wild-type cells, namely, no changes in either PI3K or pAkt/Akt were observed. AQP3-silenced SUM159PT cells did not change PI3K expression, but pAkt/Akt was significantly increased. AQP3-silenced SkBr3 cells expressed decreased PI3K after MBCD treatment, alone or with EGF, while pAkt/Akt was increased due to EGF treatment. In MCF7 cells, silencing itself decreased PI3K and increased pAkt/Akt.

## 3. Discussion

Here, we investigated whether lipid raft disruption affects AQP3 and its dependence on the EGFR/PI3K/Akt signaling pathway in breast cancer cell lines with different malignant potentials. This work focuses on AQP3 due to its involvement in breast cancer migration [13]. Breast cancer cell lines provide an interesting model to study relations between AQP3 and the EGFR signaling pathway, as MCF7 are positive for hormone receptors (ER and PR), SkBr3 are positive for human epidermal receptor 2 (HER2), and SUM159PT are negative for both hormone receptors and HER2. As it is known that EGFR can localize in lipid rafts [20,21], we performed lipid raft disruption by depleting cholesterol with MBCD [26]. Lipid raft disruption could reduce EGFR signaling, while EGF stimulates the pathway, and therefore we used a combination of both to investigate the effect on AQP3. We selected the incubation time of 2 h with MBCD, which caused no significant decrease in cell viability at a concentration of 2.5 mM. After MBCD, EGF at a concentration of 0.1 µg/mL was selected as the concentration that stimulated viability and proliferation of SkBr3 cell line in combination with MBCD. In parallel, we performed screening for aquaporin isoforms, confirming the presence of AQP3 in the cell lines. Surprisingly, the most pronounced difference was observed for AQP11, an intracellular membrane protein, which was present in cancer cell lines, but was absent in nontumorigenic cell line. This should be further investigated, as the overview of published data by Chow et. al. suggested that increased AQP11 is correlated with better prognosis in colorectal and breast cancer patients [27]. This study also found negative associations between the AQP3 gene and protein and breast cancer, indicating the need to elucidate the signaling pathways affected by AQP3 [27]. After the presence of aquaporins with peroxiporin activity was confirmed, we tested if lipid raft disruption and EGF treatment could affect this function. Although lipid rafts disruption and EGF treatment affected H_2_O_2_ uptake, this was cell-line-specific. Satooka and Hara-Chikuma showed that AQP3 is needed for CXCL12-induced cell migration by channeling H_2_O_2_, as the migratory capacity was impaired if AQP3 was knocked down [13]. This prompt us to evaluate cell migration in our breast cancer cell lines. As the closure of the wound in the wound healing assay can be the result of both cell migration and proliferation, we used mitomycin C, which blocks DNA synthesis, to inhibit proliferation so that the observed wound closure was due to migration only [28]. After mitomycin C inhibition of proliferation, we could not correlate the peroxiporin activity with the migratory capacity. Especially, as our results indicated that the triple negative cell line, SUM159PT, had migratory capacity similar to nontumorigenic cell line, MCF10A. One of the possible explanations could be that SUM159PT, being a cell line with very high proliferation rate, which was supported by the wound healing assay performed without mitomycin C (data not shown), may be therefore very sensitive to mitomycin C treatment, which would impair migration in addition to proliferation in these cells.

Our next step was to investigate the relation between the EGFR/PI3K/Akt signaling pathway and APQ3. A previous study showed that AQP3 was involved in the EGFR signaling pathway via control of H_2_O_2_ transport [12]. Even more, in gastric cancer cell lines, AQP3 overexpression upregulated matrix metalloproteinases MT MMP1, MMP2, and MMP9, while its silencing decreased them via a PI3K/Akt-dependent manner [17]. In addition, AQP3 expression is suppressed by miR-874, which directly binds to the 3′-untranslated regions of AQP3 mRNA, thereby inhibiting migration and proliferation of non-small-cell lung cancer [29], indicating multiple levels of AQP3 regulation. The localization of EGFR in lipid rafts facilitates its dimerization upon the stimuli of the receptor [20,30]. As HER2 can activate the PI3K/Akt pathway [31], we investigated whether disruption of the lipid rafts would affect EGFR signaling upon EGF stimulation by measuring the expression of downstream proteins, PI3K and Akt. In addition to Akt expression, pAkt was also examined to monitor the activation of the PI3K/Akt pathway. Effect of lipid raft disruption and EGF stimulation on PI3K/Akt, AQP3, and NRF2 expression was cell-line-specific. Therefore, we compared the effects of these treatments in nonsilenced, wild-type cell lines with AQP3-silenced cell lines. Interestingly, MCF10A did not react to either of the treatments nor to AQP3 silencing with changes in the PI3K/Akt pathway, which is in line with previously published data using an Akt inhibitor, which affected viability of triple-negative breast cancer cell lines, but not MCF10A [32]. Interestingly, lipid raft disruption and EGF stimuli increased AQP3 expression in MCF7 and SkBr3 cell lines, which can further support malignant transformation, as shown in lung adenocarcinoma, where AQP3 facilitated H_2_O_2_ uptake which further oxidized and inactivated PTEN (phosphatase and tensin homolog), inhibiting autophagy and stimulating proliferation of lung adenocarcinoma cells [33]. The triple negative cell line, SUM159PT, did not change AQP3 expression, but was the only cell line with increased NRF2 expression. These findings indicate that AQP3 could be another player in cellular oxidative response, especially due to its localization near NOX with a function of channeling H_2_O_2_ into the cell [34]. In AQP3-silenced SUM159PT cells, phosphorylation of Akt was increased, while increase in PI3K upon EGF stimuli and lipid raft disruption was ameliorated, indicating that AQP3 could be involved in the modulation of this pathway in SUM159PT. Similarly, in AQP3-silenced SkBr3 cells, an increase in pAkt was observed upon stimulation with EGF, indicating again that AQP3 modulates activity of this pathway. The fact that other peroxiporins may also be contributing to the regulation of this pathway, as in glioma and colon cancer cells where AQP5 affects the EGFR/PI3K/Akt signaling pathway [35,36], cannot be disregarded. Considering that AQP3 silencing in MCF7 cells caused decreased sensitivity to nucleotide analogs 5-FU and gemcitabine [37], while its overexpression in MCF7 spheroids decreased viability in response to cisplatin, 5-FU, and doxorubicin [38], there is a great need to investigate pathways modulated by peroxiporin(s).

Finally, our results imply that AQP3 can modulate PI3K/Akt activation and oxidative response in a cancer-cell-line-specific manner. In a hormone-positive cell line, MCF7, EGF stimuli increased phosphorylation of Akt and, in combination with lipid raft disruption, increased AQP3 expression, while AQP3 silencing decreased PI3K expression. In the HER2-positive cell line, SkBr3, migration was increased after EGF stimuli, which was not followed by changes in the PI3K/Akt pathway, while AQP3 expression was increased upon EGF stimuli in combination with lipid raft disruption. AQP3 silencing decreased PI3K expression after lipid raft disruption without any effect after EGF stimuli, while EGF stimuli increased Akt phosphorylation. In a triple-negative cell line, SUM159PT, both EGF stimuli and lipid raft disruption caused increased expression of PI3K, which was followed by an increase in Akt phosphorylation only in both treatments combined. In addition, both treatments increased NRF2 expression. AQP3 silencing did not affect PI3K expression but rather increased Akt phosphorylation, indicating that AQP3 may provide some level of regulation of this pathway in SUM159PT. In contrast to tumor cell lines, nontumorigenic breast epithelial cell line, MCF10A, did not change activity of the pathway regardless of the treatment or AQP3 silencing. Still, the exact mechanisms and possible interactions with other signaling pathways should be investigated.

## 4. Materials and Methods

### 4.1. Cell Lines

Three human breast cancer cell lines (MCF7, SkBr3, and SUM159 PT) and one human nontumorigenic epithelial breast cell line (MCF10A), purchased from EACC or Elabscience, Austria, were used in this study. MCF7 is an estrogen receptor (ER)- and progesterone receptor (PR)-positive cell line, SkBr3 is an HER2-positive cell line, and SUM159 PT is a triple-negative (ER-, PR-, and HER2-negative) cell line. Three tumor cell lines were grown in DMEM (Sigma Aldrich, St. Louis, MO, USA) supplemented with 10% FCS (fetal calf serum, Sigma Aldrich), while MCF10A was grown in DMEM:F12 1:1 (Sigma Aldrich) supplemented with 10% FCS, 20 ng/mL EGF, 10 µg/mL insulin, and 100 ng/mL cholera toxin in a humidified atmosphere with 5% CO2 at 37 °C.

### 4.2. Cell Culture Treatments

Three cancer cell lines (MCF7, SkBr3, and SUM159 PT) and one nontumorigenic cell line (MCF10A) were grown until semiconfluency, after which they were trypsinized, counted, and seeded. For MTT assay, 10,000 cells were seeded in 100 µL media and left to adhere overnight. The next day, cells were treated with 2.5 mM methyl-beta-cyclodextrin (MBCD, Carl Roth, Karlsruhe, Germany) for 2 h, after which MBCD was removed and fresh media or 0.1 µg/mL EGF were added to the cells. After 24 h, cells were either assayed for viability and proliferation or they were lysed and proteins and RNA were extracted for further analyses. The titration curves for MBCD and EGF are shown in Supplementary Materials (Figures S1–S3).

Silencing was performed with siRNA for AQP3 (SR300258, Origene, Rockville, MA, USA) and control, scrambled RNA (Darmacon, Lafayette, CO, USA), using Lipofectamine 3000 (Thermo Fisher Scientific, Waltham, MA, USA) according to the manufacturer’s instructions.

### 4.3. Viability and Proliferation Assays

After the end of the treatment, cell viability was measured by EZ4U MTT assay (Biomedica, Vienna, Austria) according to the manufacturer’s instructions. Briefly, 24 h after treatments, cells were incubated with 20 µL of the colorless dye. The dye is oxidized in the living cells to yellow water-soluble product, which is then measured on plate reader (EZ Read 2000, Biochrom, Cambridge, UK) at 450 nm, with 620 nm as a reference wavelength.

Cell proliferation was measured by BrdU assay (Roche, Basel, Switzerland), according to the manufacturer’s instructions. Briefly, 22 h after treatments, cells were incubated with 10 µL of labeling solution for 2 h. Next, cells were fixed and stained with anti-BrdU-POD solution. Color development was stopped with stop solution, and the color development was measured on a plate reader (EZ Read 2000) at 450 nm.

### 4.4. Aquaporin Activity Assay

In order to test aquaporin for H_2_O_2_ channeling, 30,000 cells were seeded on a 15 mm coverslip in 100 µL, and left for 24 h to attach. The next day, cells were treated with 2.5 mM MBCD for 2 h. After the MBCD treatment, cells were treated with 0.1 µg/mL EGF for 24 h. Before measuring the H_2_O_2_ permeability, cells were incubated with 10 µM H_2_-DCFDA for 30 min at 37 °C and 5% CO_2_. Next, the coverslips were washed with 25 mM HEPES and mounted in a perfusion chamber (Warner Instruments, Hamden, CT, USA) on a Zeiss Axiovert 200 inverted microscope. Fluorescence was excited at a wavelength of 495/10 nm using the Metafluor Software (Molecular Devices, Sunnyvale, CA, USA), which was used for data recording. Cells were equilibrated in 25 mM HEPES for 1 min, after which 100 µM H_2_O_2_ was added to cells, and fluorescence was measured every 10 s. H_2_O_2_ intake was obtained from the slope of a plot of fluorescence intensity over time and analyzed using Microsoft Excel Visual Basic Analysis code (https://github.com/nijelic/slope-residuals-for-multivariate-time-series, accessed on 19 January 2023).

### 4.5. Migration Assay

To assess the influence of lipid raft disruption and EGF stimulation on cell migration, cells were seeded in a 96-well plates at the density of 3000 cells per well and were left overnight to attach. The next day, cells were treated with 2.5 mM MBCD and 5 mg/mL mitomycin C for 2 h (titration curve shown in Supplement S4), when the cultures were scratched. The media were discarded, and fresh media or 0.1 µg/mL EGF were added to the cells. Mitomycin C was added to stop proliferation so the results of wound closure were only due to migration, and not influenced by proliferation. Cell were photographed right before scratching, and 24 and 48 h after scratching. The surface of the wound without cells was analyzed by the ImageJ software 1.53t.

### 4.6. RNA Isolation, cDNA Synthesis, PCR, and qPCR

Cells were treated as described above, and total RNA was isolated using Trizol (Thermo Fisher Scientific) according to the manufacturer’s instructions. RNA concentration and purity were assessed spectrophotometrically (NanoPhotometer^®^ N60, Implen GmbH, München, Germany), and 1 µg was used for cDNA synthesis using a High-Capacity cDNA Reverse Transcription Kit (Thermo Fisher Scientific), according to the manufacturer’s instructions.

Quantitative PCR (qPCR) was used for AQP screening and reactions were per-formed on a CFX96 RT-PCR Detection System C1000 (BioRad, Hercules, CA, USA), with TaqMan Universal PCR Master Mix (Thermo Fisher Scientific) and specific Taq-Man predesigned gene expression assays: AQP1 (Hs01028916_m1), AQP3 (Hs01105469_g1), AQP5 (Hs00387048_m1), AQP8 (Hs01086280_g1), AQP11 (Hs005426181_m1), HPRT-1 (Hs02800695_m1), and ACTB (Hs01060665_g1) (Thermo Fisher Scientific). Relative quantification of gene expression was determined with a 2–∆Ct method [39], and gene expression was normalized with two housekeeping genes (HPRT-1 and ACTB).

In order to confirm AQP3 silencing, PCR was performed on an Eppendorf 5331 MasterCycler Gradient Thermal Cycler (Eppendorf, Hamburg, Germany) using KAPA Taq PCR Kit (Sigma Aldrich) and oligonucleotide primers designed for AQP3: 5′ AG-ACAGCCCCTTCAGGATTT 3′ (AQP3-F) and 5′ TCCCTTGCCCTGAATATCTG 3′ (AQP3-R). The AQP3 PCR primers and product were validated by sequencing. PCR products were visualized after 1.5% agarose gel electrophoresis with GelGreen^®^ Nucleic Acid Stain (EMD Millipore Corporation, Temecula, CA, USA) with Alliance 4.7 Digital Imaging System (Uvitec, Cambridge, UK). Reactive bands were analyzed in Nine Alliance software and gene expression was normalized to an endogenous control (ACTB, oligonucleotide primers used in the PCR reaction: 5′ GAG-CACAGAGCCTCGCCTT 3′ (ACTB-F) and 5′ CCCACCATCACGCCCTGG 3′ (ACTB-R)). In addition, the nucleotide sequence analysis of the PCR product was conducted in order to confirm whether it was the AQP3.

### 4.7. Western Blot Analyses

Cells were treated as described above, and harvested in RIPA Buffer. Protein concentration was measured according to Bradford [40] and aliquots containing 10 µg protein were separated by SDS-PAGE on 10% resolving gel. Proteins were transferred to nitrocellulose membrane (Roti-NC 0.2 µm; Carl Roth), which was then stained with Ponceau S, scanned, and destained in distilled water. The membrane was blocked with 2% nonfat dry milk and, after removing the blocking solution, the membrane was incubated with primary antibodies (1:1000 anti-Nrf2 antibody, D1Z9C; 1:1000 anti-PI3K, C73F8; 1:1000 anti-p-Akt, D9E; 1:1000 anti-Akt, C67E7; 1:1000 anti-β-actin, D6A8, all Cell Signaling Technology (CST), Danvers, MA, SAD), and anti-AQP3 (1:200, sc-518001, Santa Cruz Biotechnology, Dallas, TX, USA). After washing, the membrane was incubated with anti-rabbit IgG, HRP-linked antibody (1:2000, 7074, CST), or anti-mouse IgG, HRP-linked antibody (1:3000, 96714, CST). The signal was visualized with SuperSignal™ West Pico PLUS Chemiluminescent Substrate (ThermoFisher Scientific) and the chemiluminescence was detected using the Alliance 4.7 Digital Imaging System (Uvitec, Cambridge, UK). Signals were quantified using the Nine Alliance software (Uvitec), and protein expression was normalized with housekeeping protein (β-actin) and Ponceau S staining.

### 4.8. Statistical Analyses

All the experiments were performed in biological and technical triplicates. Results are expressed as mean ± SEM of at least three independent experiments. Statistical analysis between groups was performed by two-way ANOVA and confirmed by the nonparametric test Mann–Whitney U-test using the Graph Prism software 8.0 (GraphPad Software, La Jolla, CA, USA); *p* values < 0.05 were considered statistically significant.

## Data Availability

The data presented in this study are available on request from the corresponding author.

## Supplementary Materials

The following supporting information can be downloaded at: https://www.mdpi.com/article/10.3390/ijms24098133/s1.
