# Supplementary material for: AQP3-Dependent PI3K/Akt Modulation in Breast Cancer Cells
Source: Int J Mol Sci. 2023 May 1;24(9):8133. doi: 10.3390/ijms24098133 (PMC10179317; doi:10.3390/ijms24098133)
Supplement: Supplementary file 1 [file ijms-24-08133-s001.zip › ijms-2245994-supplementary.pdf]

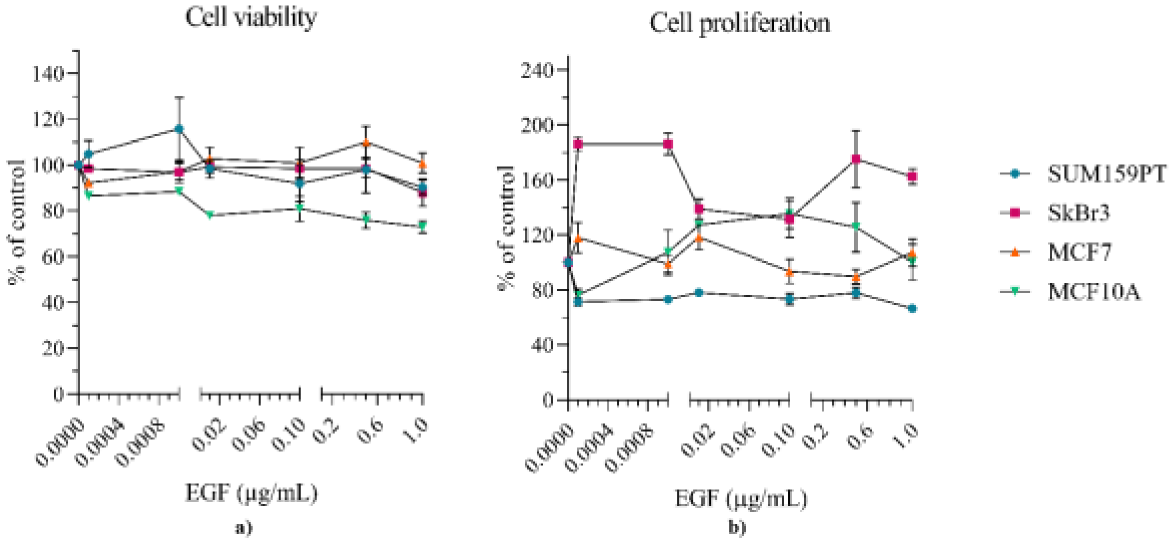

Figure S1. Cell viability (a) and proliferation (b) measurements after treatment with different concentrations of EGF**.**

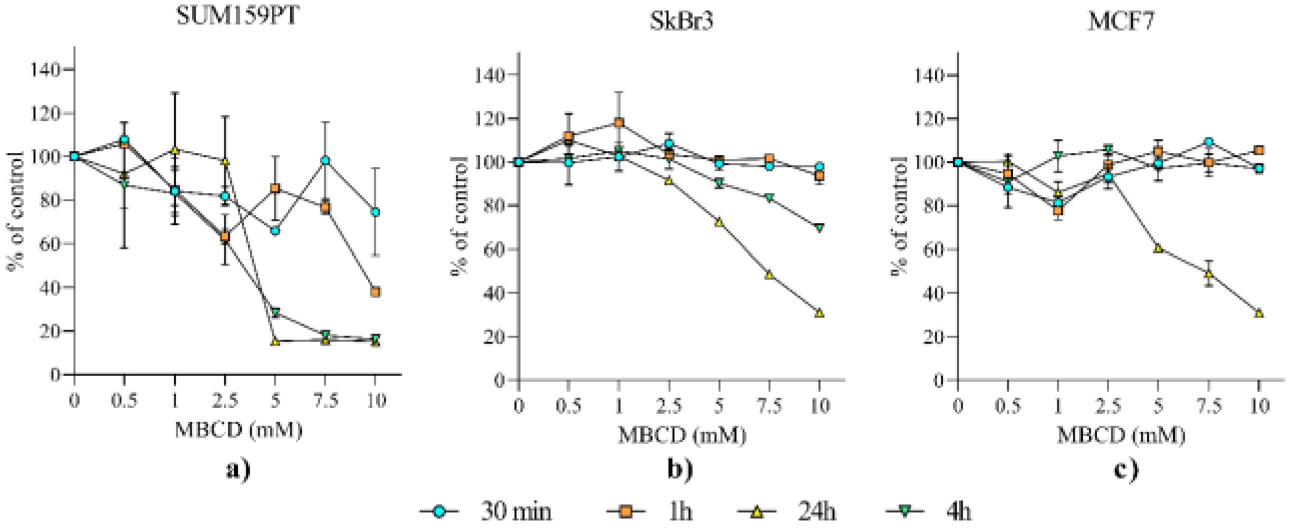

Figure S2. Cell viability measurements on SUM159PT (a), SkBr-3 (b), and MCF7 (c) after treatment with different concentrations of MBCD in different time periods.

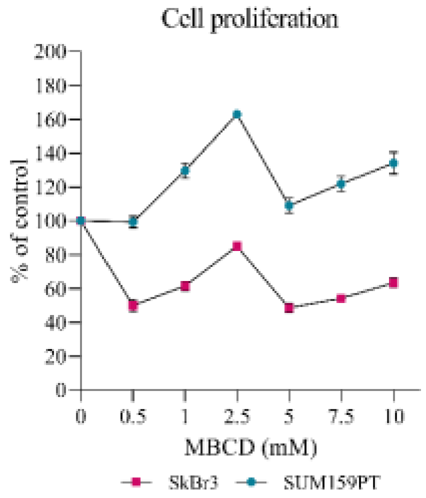

Figure S3. Cell proliferation measurements
after treatment with different concentrations of MBCD.

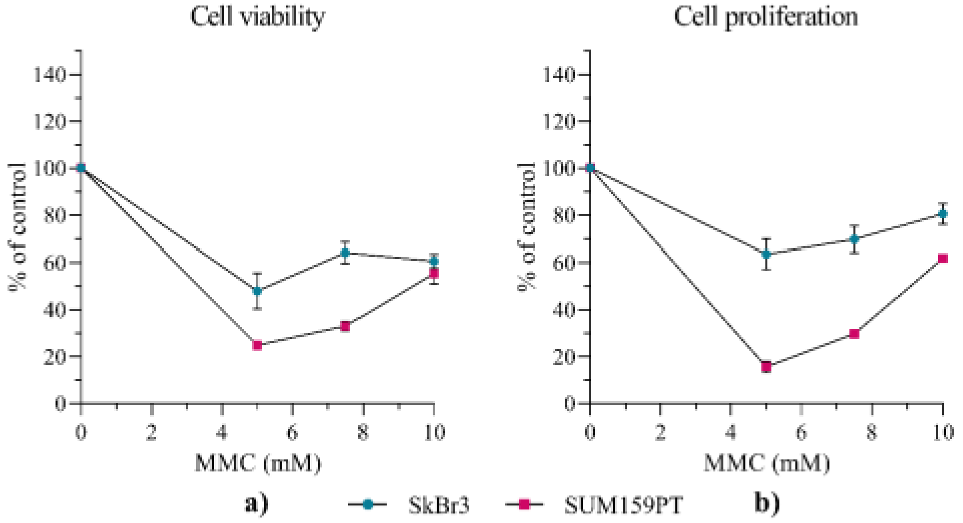

Figure S4. Cell viability (a) and proliferation (b) measurements
after treatment with different concentrations of MMC.
